# Effects of PEG Chain Length on Relaxometric Properties of Iron Oxide Nanoparticles-Based MRI Contrast Agent

**DOI:** 10.3390/nano12152673

**Published:** 2022-08-04

**Authors:** Jianxian Ge, Cang Li, Ning Wang, Ruru Zhang, Mohammad Javad Afshari, Can Chen, Dandan Kou, Dandan Zhou, Ling Wen, Jianfeng Zeng, Mingyuan Gao

**Affiliations:** 1 Center for Molecular Imaging and Nuclear Medicine, State Key Laboratory of Radiation Medicine and Protection, School for Radiological and Interdisciplinary Sciences (RAD-X), Collaborative Innovation Center of Radiological Medicine of Jiangsu Higher Education Institutions, Soochow University, Suzhou 215123, China; 2 The First Affiliated Hospital of Soochow University, Soochow University, Suzhou 215006, China

**Keywords:** iron oxide nanoparticles, relaxometric properties, PEG chain length, MRI contrast agent

## Abstract

Iron oxide nanoparticles (IONPs) as magnetic resonance imaging (MRI) contrast agents have received considerable interest due to their superior magnetic properties. To increase the biocompatibility and blood circulation time, polyethylene glycol (PEG) is usually chosen to decorate IONPs. Although the surface effect induced by the PEGylation has an impact on the relaxometric properties of IONPs and can subsequently affect the imaging results, the occurrence of particle aggregation has troubled researchers to deeply explore this correlation. To shed light on this relationship, three diphosphonate PEGs with molecular weights of 1000, 2000, and 5000 Da were used to replace the hydrophobic oleate ligands of 3.6 nm and 10.9 nm IONPs. Then, the contrast enhancement properties of the resultant “aggregation-free” nanoparticles were carefully evaluated. Moreover, related theories were adopted to predict certain properties of IONPs and to compare with the experimental data, as well as obtain profound knowledge about the impacts of the PEG chain length on transverse relaxivity (*r*_2_) and longitudinal relaxivity (*r*_1_). It was found that *r*_2_ and the saturated magnetization of the IONPs, independent of particle size, was closely related to the chain length of PEG. The results unveiled the correlation between the chain length of the coated PEG and the relaxometric properties of IONPs, providing valuable information which might hold great promise in designing optimized, high-performance IONPs for MRI-related applications.

## 1. Introduction

Owing to their superior magnetic and biocompatible properties, iron oxide nanoparticles (IONPs) have been widely used for biomedical applications such as magnetic resonance imaging (MRI) [1,2,3], drug delivery [1], cell tracking [4,5], and gene therapy [6,7]. In particular, IONPs serve as contrast agents for the MRI, offering noninvasive and real-time manners to visualize the anatomical structure of the body. Therefore, they have received remarkable interest in single-mode MRI [8,9,10], multimodality imaging such as PET/SPECT-MRI [11,12], MRI-CT [13,14], and MRI-optical imaging [15,16], as well as MRI-guided therapy such as MRI-PTT/PDT [17,18] and MRI-chemotherapy [19]. To achieve better MRI diagnosis accuracy, a great number of efforts have been devoted to improve the contrast enhancement effect of IONPs in the last two decades.

As one of the most important parameters of contrast agents, relaxivity provides a valuable measure for evaluating the degree with which the material can highlight the contrast between the tissue of interest and its surrounding areas. Since a higher relaxivity corresponds to a better contrasting capability, a large number of studies were carried out to obtain contrast agents with enhanced relaxivity through tailoring different characteristics of the materials. Thus far, it is well-accepted that the relaxivity of IONPs depends on many factors such as particle size and size distribution [20,21,22], surface properties [23,24,25,26], state of aggregation [10,27,28,29,30], and shape [31,32]. Among the factors mentioned above, the size effect is the most intensively investigated parameter, and a set of size-related criteria was suggested for comprehensive tuning of the relaxivity and pharmacokinetics of IONPs. Moreover, the surface modification of nanoparticles plays an important role in their biomedical applications. Particularly, proper surface modification of IONPs can ensure their colloidal stability as well as capability to effectively escape immunological attacks following administration in blood, resulting in a long blood residence time. To date, many kinds of materials such as DMSA [33], PEG [34,35,36], PVP [37], and silica [38,39] have been employed as the surface coating layer of IONPs for MRI applications, among which PEG has shown undoubted advantages in constructing biocompatible probes. Although some efforts have been made to gain insight into the effect of surface chemistry on the contrast properties of IONPs [24,32,40,41], the influence of surface coating on the relaxivity of the nanoparticles has not been well understood. The particle aggregation, which usually occurs following the surface modification, has troubled researchers to identify the relationship between the relaxometric properties and the surface chemistry of IONPs. In our previous study, taking advantage of PEG derivatives with different anchoring moieties including diphosphonate, catechol, and hydroxamate groups, which have high affinity towards IONPs, the aggregation formation of the nanomaterials was hindered [25]. Additionally, a plausible explanation for the effect of anchoring groups on the relaxivity of IONPs was suggested. However, with respect to the PEG chain length, most of the studies focused on the impacts on the pharmacokinetics and biodistribution [42,43]. The effects on the relaxometric properties of IONPs remained unclear and need to be explored in-depth.

Therefore, the current study was devoted to systematically investigate the effect of surface modification on the relaxivity of IONPs. Building upon our previous study, diphosphonate-PEG (DP-PEG) derivates with different molecular weights were employed to replace the native hydrophobic ligands on 3.6 nm and 10.9 nm-sized IONPs. Due to the strong binding ability of the diphosphonate group to IONPs, two differently sized hydrophilic probes with varying PEG chain length were obtained. The resulting hydrophilic IONPs can individually disperse in aqueous solution, eliminating the disturbance of aggregated nanoparticles as a consequence. Thus, the prepared samples served as great candidates to take part in the investigation of PEG length effect on the relaxometric properties of IONPs, providing valuable information for designing high-performance nano-contrast agents for MRI applications.

## 2. Materials and Methods

### 2.1. Materials

Ferric acetylacetonate (Fe(acac)_3_) was purchased from Sigma–Aldrich and used after two recrystallizations. Diphenyl ether was used after vacuum distillation. Oleic acid, oleylamine, 1-octadecene, and 1-octadecanol were purchased from Sigma–Aldrich and used as received. Other chemicals of analytical grade, including ethanol, ether, cyclohexane, and tetrahydrofuran (THF), were used as received. Diphosphonate PEG (*M*_w_ ≈ 1000, 2000, and 5000 Da) with a diphosphonate group at one end of the chain and a methoxy group at the other, were customized products provided by Suzhou Xinying Bio-Medical Technology Co. Ltd. (Suzhou, China) The iron oleate complex was prepared according to a previous report [42].

### 2.2. Synthesis of Hydrophobic IONPs 

IONPs with a core size of 3.6 nm were synthesized according to a previous report, with slight modifications [2]. Typically, 1.41 g (4 mmol) of Fe(acac)_3_, 3.39 g (12 mmol) of oleic acid, 3.21 g (12 mmol) of oleylamine, and 2.70 g (10 mmol) of 1-octadecanol were dissolved in 40 mL of diphenyl ether. After being purged with nitrogen for 30 min, the solution was refluxed for 30 min under stirring. Then, the reaction system was cooled to room temperature. The resultant nanoparticles were precipitated by ethanol, collected by centrifugation, washed with ethanol three times, and finally, redispersed in THF or cyclohexane for further experiments.

IONPs with a core size of 10.9 nm were also synthesized according to a previous report [1]. In brief, 3.6 g (4 mmol) of freshly prepared iron oleate and 3.39 g (4 mmol) of oleic acid were dissolved in 25 mL of 1-octadecene. The resultant solution was heated to 310 °C at a rate of 3.3 °C/min and then maintained at 310 °C for 30 min under nitrogen protection. The preparation was terminated by cooling the reaction mixture to room temperature. The solution was subjected to the purification steps mentioned above for 3.6 nm nanoparticles and resulted in obtaining 10.9 nm IONPs.

### 2.3. Ligand Exchange

Typically, 150 mg of PEG derivative was dissolved in 10 mL of THF containing 10 mg hydrophobic IONPs. Then, the reaction mixture was heated to 60 °C and kept at this temperature for 12 h under stirring. Afterwards, the IONPs were precipitated by cyclohexane, isolated via centrifugation, washed with cyclohexane three times, and then dried under a vacuum at room temperature. The particle powders obtained in this way, independent of the molecular weight of PEG and the particle core size, were found to be readily dissolved in water, supporting that the PEG coating was effectively realized. To remove excess PEG ligands, the resultant aqueous solutions containing the PEGylated IONPs were purified by ultrafiltration for 4 cycles using a 100 kDa MWCO centrifugal filter (Millipore YM-100, Merck, Germany).

### 2.4. Characterizations

Transmission electron microscopy (TEM) images of the nanoparticles were taken on a JEM-100CXII electron microscope (JEOL, Japan) at an acceleration voltage of 100 kV. The particle size was determined by averaging at least 300 particles per sample. Powder X-ray diffraction (XRD) patterns of the particle samples were recorded on a D/Max-2500 diffractometer (Regaku, Japan) under Cu K*α*_1_ radiation (*λ* = 1.54056 Å). TGA measurements were performed on a TG209F3 thermogravimetric analyzer (NETZSCH, Germany). The magnetic properties of the resultant samples were characterized by using a vibrating sample magnetometer (VSM JDM-13, Changchun Yingpu Magnetoelectric Technology Development Co., Ltd., China). Dynamic light scattering (DLS) measurements were carried out at 298.0 K with a Nano ZS (Malvern, United Kingdom) equipped with a solid-state He-Ne laser (*λ* = 633 nm) for measuring the hydrodynamic size of the resultant nanoparticles. The concentration of Fe was determined by using an inductively coupled plasma atomic emission spectrometer (ICP-2000) produced by Jiangsu Skyray Instrument Co., Ltd. (China)

### 2.5. Relaxivity Measurements

The relaxivity measurements were carried out on a 3 T clinical MRI instrument (Signa 3.0 T HD, GE, Milwaukee, WI, USA). A series of aqueous solutions of PEG-coated IONPs in 2 mL Eppendorf tubes were prepared. The parameters for *T*_1_ measurements were set as follows: echo time (TE) = 25.3 ms and repetition time (TR) = 500, 1000, 1500, and 2000 ms. For *T*_2_ measurements, the parameters were set as TR = 2000 ms and TE = 20, 40, 60, 80, and 100 ms.

## 3. Results and Discussion

### 3.1. Synthesis of Hydrophobic IONPs 

**Figure 1:**
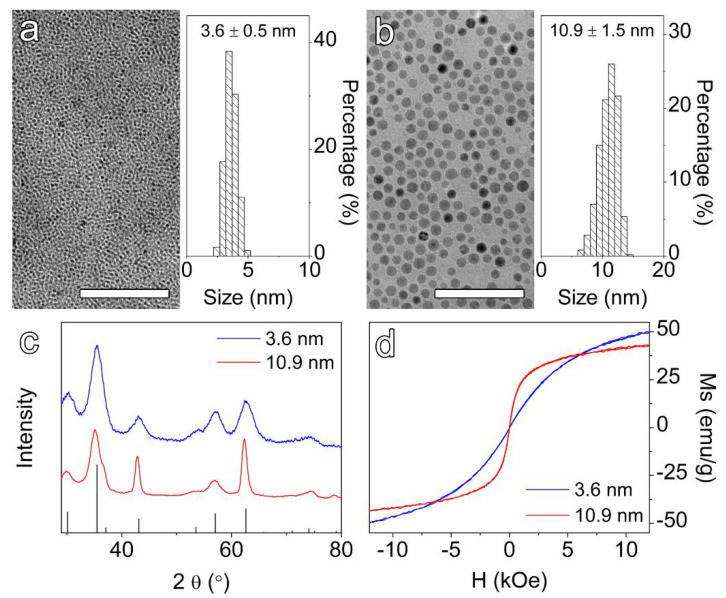
TEM images and histograms of the as-prepared hydrophobic 3.6 nm (**a**) and 10.9 nm (**b**) IONPs. (**c**) X-ray diffraction patterns of the hydrophobic samples together with the JCPDS card data (JCPDS 88-0866) for magnetite shown at the bottom. (**d**) Room-temperature magnetization curves of hydrophobic IONPs. The scale bars correspond to 50 nm.

The hydrophobic IONPs were synthesized based on the thermal decomposition method according to previous reports, with slight modifications [44,45]. The representative TEM images of the as-prepared nanoparticles together with their corresponding particle size distribution profiles are shown in Figure 1a,b. As it can be seen from the figures, nearly monodispersed particles with narrow size distributions and average particle sizes of 3.6 ± 0.5 nm and 10.9 ± 1.5 nm were obtained. For the sake of simplicity, the former samples are referred to as “small” and the latter ones are labeled as “large” nanoparticles throughout this manuscript. The X-ray diffraction patterns of both the small and large nanoparticles shown in Figure 1c matched well with that of the magnetite. Moreover, the room-temperature magnetization curves illustrated in Figure 1d implied that these two groups of samples were superparamagnetic. However, the magnetization (*M*_s_) did not reach the state of saturation for either sample while measured under a range of magnetic fields from the weakest to the highest available in our equipment (12 kOe), suggesting that these two differently sized samples were also prone to paramagnetism. In addition, due to a higher degree of spin disorder on their particle surfaces, the small particles displayed stronger paramagnetic behavior. Although the magnetizations recorded at 12 kOe (i.e., 50.0 emu/g for the small and 42.6 emu/g for the large IONPs) were not fully saturated, they were considered as quasi-saturated magnetizations in interpretation of their performances in MR contrast enhancement effects.

### 3.2. Ligand Exchange of Hydrophobic IONPs

Thermal decomposition of metal precursors at high temperature in the presence of oleic acid and/or oleylamine in organic media has become the most successful approach for producing monodispersed IONPs. However, the as-prepared IONPs are hydrophobic and cannot be directly used for biomedical applications. As the phosphate group has a strong binding affinity with IONPs, DP-PEG was employed to exchange the hydrophobic ligands on the particle surface and render the hydrophobic IONPs water-soluble. In order to investigate the effect of PEG chain length on the MR contrast enhancement effect, three different molecular weights of DP-PEG, namely 1, 2, and 5 kDa, were utilized. Depending on the molecular weight and the size of the IONPs (small or large), the resulting six hydrophilic samples were labeled as S/L-DP-1/2/5K (e.g., S-DP-1K refers to the samples prepared after exchanging the PEG of 1 kDa molecular weight with the native ligands of small IONPs). After the ligand exchange process, the nanoparticles were dispersed in water, forming transparent and aggregation-free solutions (Figure S1 in Supporting Information).

**Figure 2:**
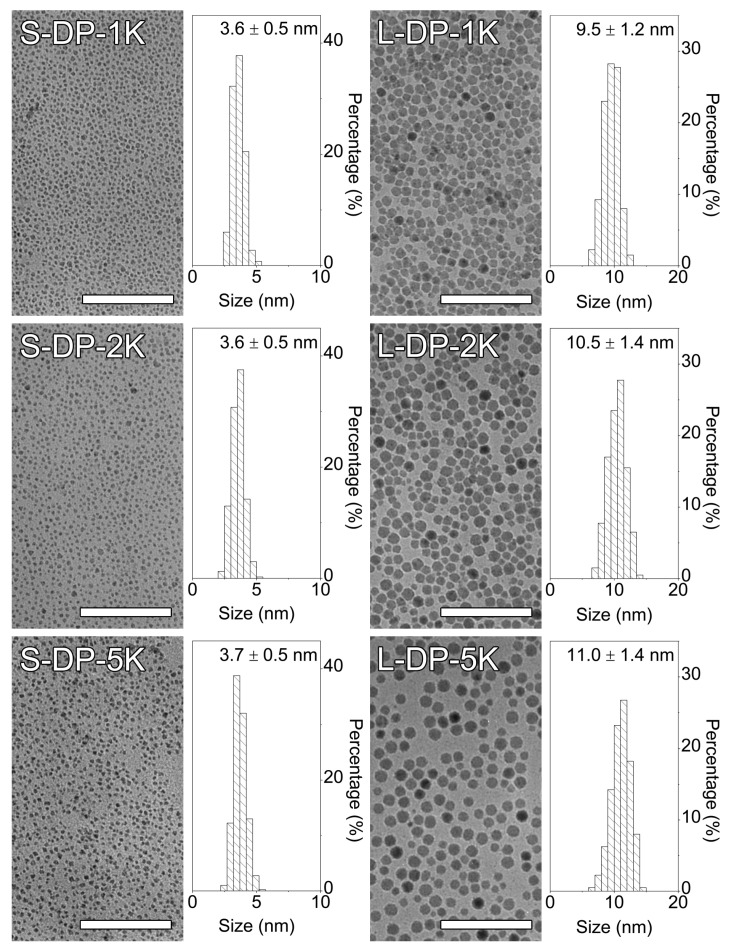
TEM images and histograms of the PEGylated IONPs after ligand exchange. The scale bars correspond to 50 nm.

To gain further insights into the impact of ligand exchange on the morphology of the PEGylated IONPs, TEM measurements were carried out thoroughly, and the detailed results are provided in Figure 2. As suggested by the results, particle aggregation was observed in neither of the six resultant PEGylated samples. Statistical results on particle size indicated that excluding the large IONPs, which encountered a size reduction from 10.9 to 9.5 nm following producing L-DP-1K, the ligand exchange process did not alter the average size and size distribution profiles of the other five samples. The reason for the size reduction in the case of L-DP-1K might lie in the fact that a fixed mass ratio of PEG to IONPs was adopted in the ligand exchange processes. Since PEG with a low molecular weight of 1 kDa was used in producing the L-DP-1K sample, excessive amounts of PEG slightly etched the iron oxide particles. Nevertheless, such phenomenon was not observed for the large IONPs treated by the 2 and 5 kDa PEGs (i.e., L-DP-2K and L-DP-5K sample, respectively).

**Figure 3:**
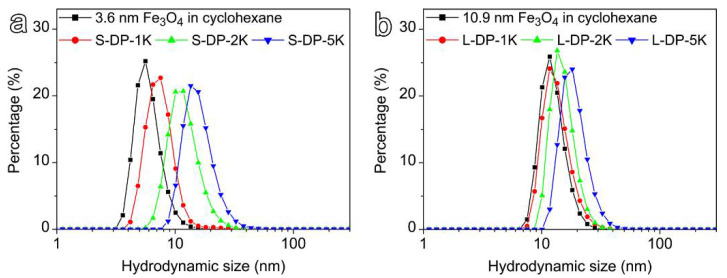
Number weighted hydrodynamic size profiles of 3.6 nm (**a**) and 10.9 nm (**b**) IONPs capped by hydrophobic ligands or DP-PEG of different chain lengths. The small (3.6 nm) and large (10.9 nm) IONPs have been denoted as S and L, respectively. Diphosphonate PEGs (DP) with molecular weights of 1, 2, and 5 kDa were labeled as DP-1K, DP-2K and DP-5K, respectively.

Although the resultant PEGylated particles in powder form could spontaneously be dissolved in water, any form of aggregation would alter the relaxometric properties of the samples and consequently, pose major obstacles for extracting magnetic relaxivity related to particle size and surface coating structures. Therefore, dynamic light scattering analysis was carried out to characterize the solution properties of the resultant IONPs. As shown in Figure 3, the small and large IONPs exhibited relatively narrow particle size distributions in cyclohexane (before ligand exchange), displaying single, scattering peaks located at 6.1 nm and 12.6 nm, respectively. After ligand exchange, the hydrodynamic size of all the PEGylated nanoparticles increased by different degrees, while the size distribution profiles remained nearly unchanged in comparison to those of their hydrophobic counterparts. In addition, the hydrodynamic size gradually increased with the PEG chain length for both the small and large IONPs. Moreover, the experimentally determined hydrodynamic sizes were in good agreement with the theoretical predictions based on an empirical formula provided in the literature [46], further indicating that the PEGylated particles were homogeneously dispersed in water with no aggregations being present. In addition, all samples possessed excellent colloidal stability and no precipitate was observed even after being stored for 3 years. The lack of aggregation is of great importance for investigating the contrast effects associated with particle size and surface chemistry. More detailed DLS measurement results are given in Table S1 and Figure S2.

### 3.3. MRI Enhancement Effects of the PEGylated IONPs

**Figure 4:**
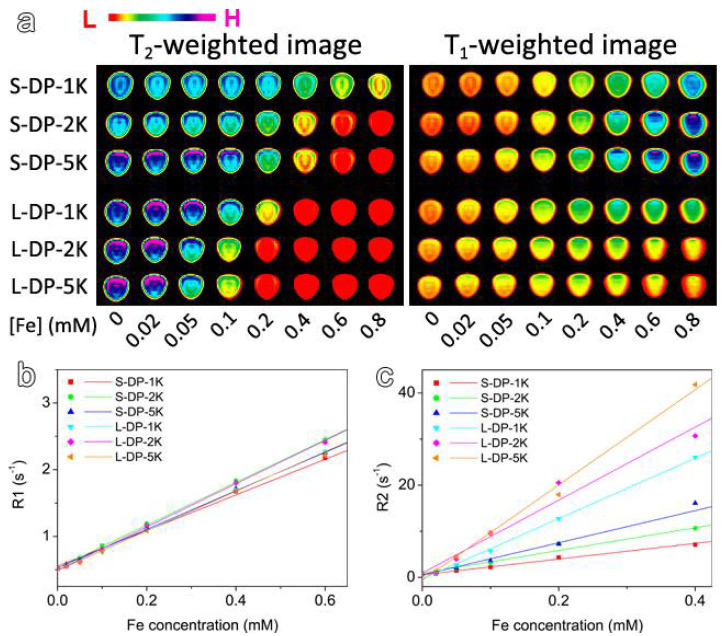
T2- and T1-weighted MR images of IONPs capped with DP-PEG of different chain lengths (**a**). Plot of R1 (**b**) and R2 (**c**) versus Fe concentration of IONPs capped with DP-PEG of different PEG chain lengths.

To evaluate the MR enhancement effect of the various types of PEGylated IONPs mentioned above, relaxivity measurements were performed on a clinical 3 T MRI scanner at room temperature. Figure 4a shows the *T*_1_- and *T*_2_-weighted MR images of the small and large IONPs capped with DP-PEG, with different PEG chain lengths. As expected, small IONPs showed obvious *T*_1_ and *T*_2_ contrast effects, especially when the concentration of iron ions ([Fe]) was higher than 0.6 mmol/L. In contrast, the large IONPs exhibited a stronger *T*_2_ contrast effect and no obvious *T*_1_ effect. The concentration-independent longitudinal (*r*_1_) and transversal (*r*_2_) relaxivities are important parameters for qualifying MRI contrast agents in practice. Therefore, obtaining a comprehensive understanding of the structural impacts of PEGylated IONPs on *r*_1_ and *r*_2_ is of great significance. These parameters were extracted through linear regression fitting of the plots of experimentally determined longitudinal and transverse relaxation rates of water protons against the [Fe], as shown in Figure 4b,c. The fitting results listed in Table 1 indicate that all the samples exhibited comparable *r*_1_ values but higher *r*_2_ values in comparison to the clinical Gd-based contrast agents [47,48]. In addition, regardless of the PEG chain length, large IONPs showed higher *r*_2_ values compared to the small IONPs. Moreover, IONPs of both sizes exhibited monotonically increased *r*_2_ against the PEG chain length, contrasting with the slight variation and nonmonotonic behaviors of *r*_1_, which showed a maximum value for the samples coated with 2 kDa PEG.

Theoretically, the relaxation enhancement of IONPs is mainly dominated by an outer-sphere mechanism, which originates from the relaxation enhancement of water molecules in bulk water. Under a high magnetic field, the *r*_2_ and *r*_1_ of IONPs can be expressed by [25,49,50,51,52]:

(1) $$r_{2}=\frac{256\pi^{2}\gamma_{I}^{2}M_{n}}{1215\rho }{\left(\frac{1}{1+L/a}\right)}^{3}M_{s}^{2}\tau_{D}$$

(2) $$r_{1}=\frac{128\pi^{2}\gamma_{I}^{2}M_{n}}{405\rho }{\left(\frac{1}{1+L/a}\right)}^{3}M_{s}^{2}\tau_{D}J_{A}\left(\sqrt{2\omega_{I}\tau_{D}}\right)$$

where *γ*_I_ is the proton gyromagnetic ratio and *M*_S_ is the saturation magnetization of the IONP core. *M*_*n*_ and *ρ* are the molar mass and bulk phase density of IONPs, respectively; *a* is the radius of the IONP core; *r* stands for the effective radius of particles which is equal to *a* plus the water impermeable thickness (*L)* of the surface coating layer (*r* = *a* + *L*); *τ*_*D*_ is the translational diffusion time (*τ*_*D*_ = *r*^2^/*D*, *D* is the water translational diffusion constant); *ω*_I_ is the proton Larmor frequency; and *J*_A_ represents Ayant’s spectral density function that can be given by:

(3) $$J_{A}\left(z\right)=\frac{1+\frac{5z}{8}+\frac{z^{2}}{8}}{1+z+\frac{z^{2}}{2}+\frac{z^{3}}{6}+\frac{4z^{4}}{81}+\frac{z^{5}}{81}+\frac{z^{6}}{648}}$$

According to Equation (1), the transverse relaxivity of IONPs depends on many factors, including the particle core size, saturation magnetization, water impermeable thickness of the surface coating layer, and translational diffusion time of protons. As demonstrated previously, the hydrophilic PEGs were effectively replaced with hydrophobic ligands on the particle surface based on their higher binding affinities to iron, which would result in negligible water impermeable thickness of the surface coating layer (i.e., *L* ≈ 0). Therefore, for IONPs with the same core size, the transverse relaxivity is mainly related to the saturation magnetization and translational diffusion time.

Table 2 and Figure S3 show the VSM results of IONPs coated with DP-PEGs of different PEG lengths. The saturation magnetizations of the samples generally decreased after ligand exchange, regardless of the PEG chain length. This can be attributed to the enhanced disturbance of the surface spin disorder layer thickness caused by PEGylation of the particle surface. In addition, it is worth noting that the saturation magnetization increased with increasing PEG chain length. Presumably, PEG derivatives of longer chain lengths caused higher steric hindrance, giving rise to relatively weaker bonding activity to iron on the particle surface, which in turn, might lead to a thinner surface spin disorder layer. This conjecture is supported by our previous report in which the saturated magnetization of IONPs was shown to be inversely correlated to the binding affinity of PEG derivates capped on the surface of nanoparticles [25].

In comparison with the saturation magnetization, the translational diffusion time is immeasurable. However, according to the expression of *τ*_D_, it can be easily inferred that the permeability of the PEG coating layer to water molecules can prolong the translational diffusion time because the diffusivity of water molecules in the PEG coating layer is lower than that of water in the bulk phase. Obviously, the translational diffusion time is dependent on the PEG chain length and density on the particle surface. In other words, the longer the PEG chain length is or the higher the PEG density is, the longer the translational diffusion time will be. As DP-PEG of longer chain length exhibit relatively weaker bonding activity to iron on the particle surface, IONPs coated with the longer chain lengths of the polymer would undoubtedly have lower PEG density on their surface. Thus, since the chain length and density have antagonistic effects, evaluating the translational time is difficult.

Nevertheless, as shown in Table 1, the transverse relaxivity was directly proportional to the PEG chain length, which was consistent with the saturation magnetization trend, indicating that the transverse relaxivity difference was dominated by the variation in saturation magnetization. This observation seems reasonable, since the transverse relaxivity is proportional to the translational diffusion time and the square of saturation magnetization.

Similar to the case of transverse relaxivity discussed above, the longitudinal relaxivity is also proportional to the square of saturation magnetization. However, as shown in Figure S4, since the term of $\tau_{D}J_{A}\left(\sqrt{2\omega_{I}\tau_{D}}\right)$ in Equation (2) is nonmonotonic for *τ*_D_, this proportionality is more complex compared to that of transverse relaxivity. The value of the *τ*_D_*J*_A_ term rises at the beginning, then drops with the increase in translational diffusion time. As mentioned previously, the relative order of the translational diffusion time for IONPs coated with DP-PEG of different chain lengths is unpredictable. Therefore, the evaluation of the longitudinal relaxivity change caused by the translational diffusion time is intricate. Nevertheless, the results showed that the variation in both of the saturation magnetization and translational diffusion time did not induce any noticeable change in the longitudinal relaxivity of IONPs, with respect to the PEG chain length.

Depending on the application of interest, specific optimizations of certain properties of IONPs need to be performed. For instance, striking a balance between the pharmacokinetics and relaxometric properties of IONPs is necessary for tumor diagnosis in order to prepare high performance nanoprobes. According to previous reports, PEG chain length has remarkable effects on the blood circulation time and metabolic behaviors of IONPs [43,53]. In the current work, it was shown that the PEGylation of IONPs would also result in a relaxivity change of IONPs. We believe that the results of our study shed light on the correlation between these important parameters, providing essential information for seeking the balance.

## 4. Conclusions

In summary, diphosphonate PEGs with molecular weights of 1, 2, and 5 kDa were used to exchange the hydrophobic ligands of 3.6 nm and 10.9 nm IONPs. The high binding affinity of diphosphonate groups for Fe^3+^ ions enabled a reliable ligand exchange process for achieving PEGylated IONPs that were well dispersible in water. As a result of the absence of particle aggregation in the resultant solutions, in-depth evaluations of the impacts of the PEG chain length on the contrast effects of the underlying IONPs became feasible. Further systematic investigation showed that the transverse relaxivity, *r*_2_, was positively correlated with the PEG chain length due to the enhanced saturation magnetization caused by increasing in PEG chain length. Conversely, the longitudinal relaxivity, *r*_1_, exhibited slight and non-monotonous variation with changing PEG chain length, which might be attributed to the antagonistic effects of translational diffusion time and saturation magnetization. In conclusion, the current investigations disclose the impacts of the chain length of PEG ligands on the relaxometric properties of the underlying IONPs and thus, provide valuable information for constructing high-performance MRI contrast agents for specific applications.

## Figures and Tables

**Table 1:** Effects of PEG chain length on the relaxivities of DP-PEG-capped IONPs.

3.6 nm IONPs			10.9 nm IONPs		
Sample	*r*_2_ (mM^−1^ s ^−1^)	*r*_1_ (mM^−1^ s ^−1^)	Sample	*r*_2_ (mM ^−1^ s ^−1^)	*r*_1_ (mM^−1^ s ^−1^)
S-DP-1K	16.97	2.67	L-DP-1K	65.37	2.96
S-DP-2K	24.64	3.21	L-DP-2K	79.07	3.24
S-DP-5K	34.82	2.89	L-DP-5K	103.28	2.97

**Table 2:** The saturation magnetization of IONPs coated with DP-PEGs of different chain lengths ^1^.

3.6 nm IONPs		10.9 nm IONPs	
Sample	*M*_S_ (emu/g)	Sample	*M*_S_ (emu/g)
S-DP-1K	7.69	L-DP-1K	11.69
S-DP-2K	12.97	L-DP-2K	19.79
S-DP-5K	17.99	L-DP-5K	28.13

^1^ The saturation magnetization refers to the quasi-saturated magnetization recorded at 12 kOe. The magnetization was normalized to the mass of IONPs (the PEG mass was subtracted based on TGA results).

## Supplementary Materials

The following are available online at https://www.mdpi.com/article/10.3390/nano12152673/s1, Figure S1: Photographs of PEGylated IONPs in aqueous solution, Figure S2: Detailed DLS measurement results of large IONPs, Figure S3: Room-temperature magnetization curves of PEGylated IONPs, Figure S4: The *τ*_D_*J*_A_ profile computed under magnetic field of 3 T, Table S1: The experimental and predicted hydrodynamic sizes of the PEGylated IONPs.
