# Supplementary material for: Effects of PEG Chain Length on Relaxometric Properties of Iron Oxide Nanoparticles-Based MRI Contrast Agent
Source: Nanomaterials (Basel). 2022 Aug 4;12(15):2673. doi: 10.3390/nano12152673 (PMC9370369; doi:10.3390/nano12152673)
Supplement: Supplementary file 1 [file nanomaterials-12-02673-s001.zip › nanomaterials-1822693-supplementary.pdf]

Supporting Information

Effects of PEG Chain Length on Relaxometric Properties of Iron Oxide Nanoparticles-Based MRI Contrast Agent

Table S1. The experimental and predicted hydrodynamic sizes of the PEGylated IONPs.

Sample	Experimental hydrodynamic size[a] (nm)	PDI[a]	Predicted hydrodynamic size[b] (nm)
S-DP-1K	7.7±1.2	0.51±0.11	7.2
S-DP-2K	12.2±1.5	0.51±0.10	9.0
S-DP-5K	16.3±1.4	0.44±0.03	12.6
L-DP-1K	13.9±0.5	0.16±0.01	13.1
L-DP-2K	15.3±0.2	0.35±0.02	15.9
L-DP-5K	19.2±0.3	0.29±0.01	19.9

[a] Average results of four measurements. [b] The predicted hydrodynamic size was obtained by assuming a simple addition of the core size and two times the calculated hydrodynamic diameter of the PEG ligands ($d_{PEG} = 0.03824 M_n^{0.559}$).

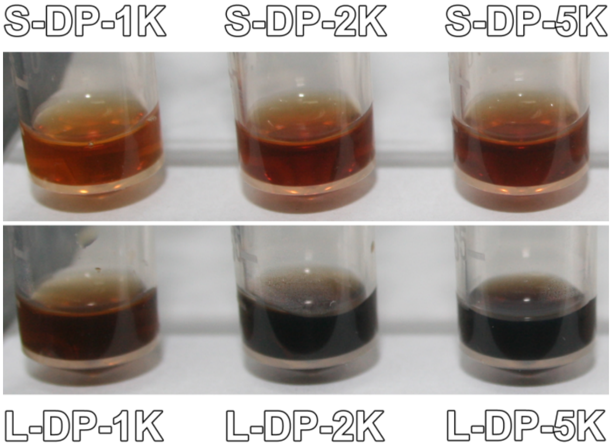

Figure S1. Photographs of PEGylated IONPs in aqueous solution.

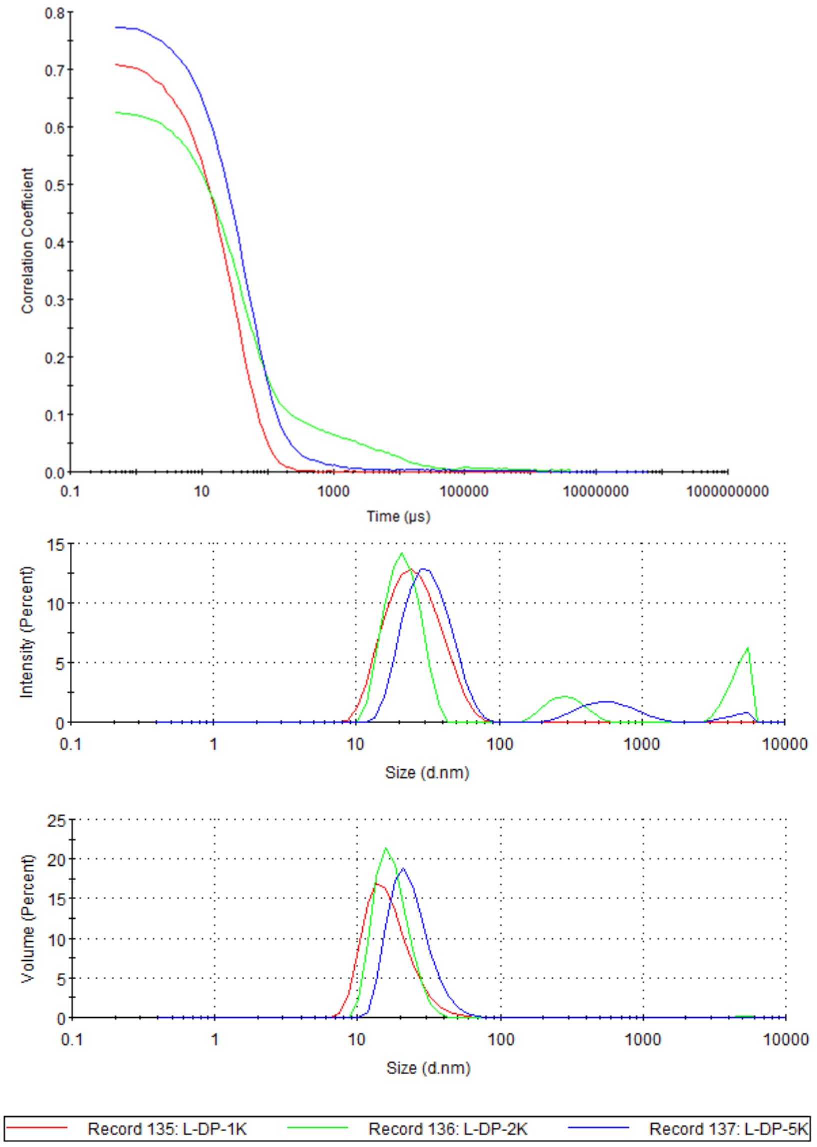

Figure S2. The typical correlograms (upper frame), size distributions by intensity (middle frame), and size distributions by volume (buttom frame) of large IONPs.

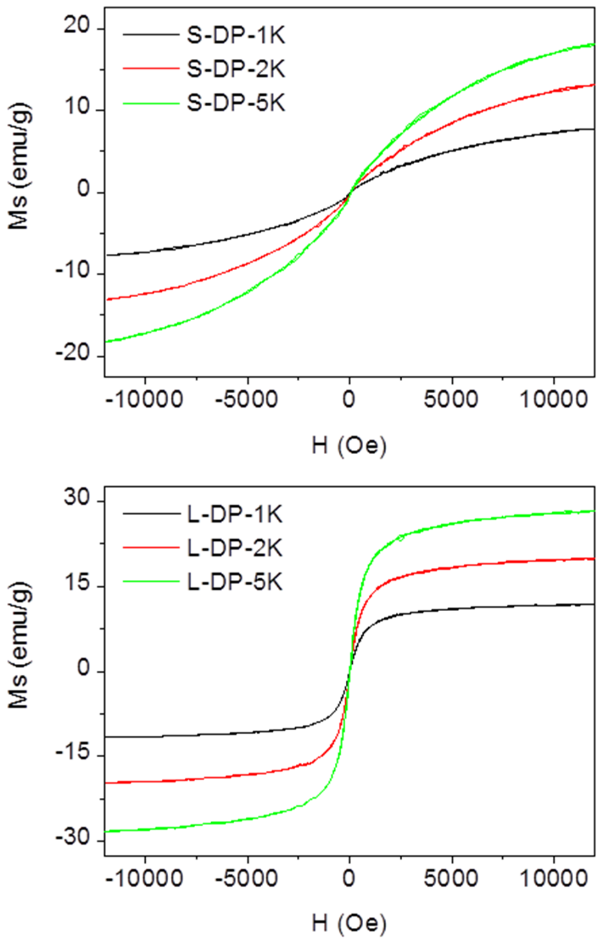

Figure S3. Room-temperature magnetization curves of PEGylated IONPs.

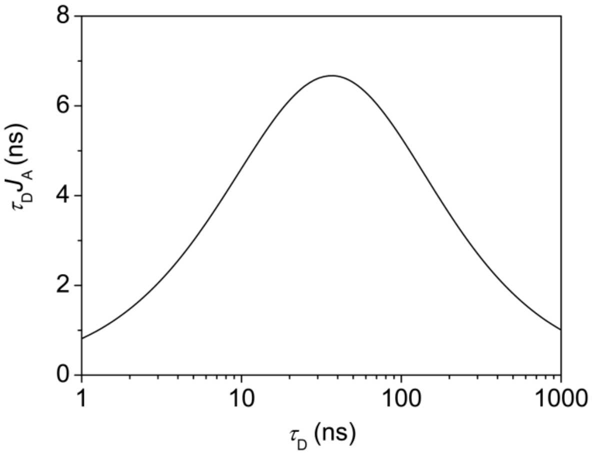

Figure S4. The $\tau_D J_A$ profile computed under magnetic field of 3T.
